# 

**Published:** 1920-09

**Authors:** 


					*L.
SEPTEMBER, 1920.
v?l XXXVIL No. 140,
THE
BRISTOL
medico-chiru rg u
f NOV 2 1820
Editor: P. WATSON-WILLIAMS, M.D.,
WITH WHOM ARK ASSOC1AT1CD
JAMES SWAIN. C.B., C.B.E., M.S., M.D.,
J. LACV FIRTH, M.S., M.D., CAREY COOMBS, M.D.
J. A. NIXON, C.M.G., M.D., Assistant Editor.
Editorial Secretary: J. M. FORTESCUE-BRICKDALE, M.A., M.D.
BRISTOL: J. W. ARROWSMITH LTD.
London : Simpkin, Marshall, Hamilton, Kent & Co. Ltd.
Price Three Shillings net.

				

